# Online Racism, Digital Mental Health Tools, and Online Mental Health Communication Among Black Young Adults With and Without Depression or Anxiety: Cross-Sectional Quantitative Study

**DOI:** 10.2196/80657

**Published:** 2026-01-20

**Authors:** Melissa Christine Holland, Kyle Aaron Walker, Oreoluwa Oluwatomisin Badejoh, Vanessa Victoria Volpe, Brian TaeHyuk Keum, Jimi Huh, Hans Oh

**Affiliations:** 1 Department of Psychology, North Carolina State University, Raleigh, NC, United States; 2 School of Public Health, University of California, Berkeley, 2121 Berkeley Way, Berkeley, CA, 94704, United States, 1 (213) 821-1382; 3 Keck School of Medicine, University of Southern California, Los Angeles, CA, United States

**Keywords:** digital health, digital mental health, mental health, young adults, racism, depression, anxiety

## Abstract

**Background:**

Use of technological resources that provide support for mental health (ie, digital mental health tools) and opportunities to use the internet to communicate with others or receive information about mental health (ie, online mental health communication) are growing in popularity among young adults (aged 18‐29 y). However, whether exposure to the negative experience of racism online is associated with the use of digital mental health tools and online mental health communication remains an important empirical question for Black young adults, given their frequent online use and engagement.

**Objective:**

This study sought to examine (1) how the frequency of exposure to online racism is associated with the use of digital mental health tools and engagement in online mental health communication and (2) how these associations differ for Black young adults with either anxiety and depression versus those without.

**Methods:**

Conducted from July to September 2024, data came from a larger cross-sectional study of 1005 monoracial Black young adults (mean age 24.07, SD3.04 y; 50.6% women) who completed an online survey and self-reported measures of exposure to online racism, use of digital mental health tools, frequency of engaging in online mental health communication, and anxiety and depressive symptoms. Two separate path analysis regression models were conducted for the outcomes of depression and anxiety.

**Results:**

Our results showed that more frequent exposure to online racism was associated with a greater likelihood of using digital mental health tools (odds ratio [OR] range 1.72‐1.84; *P*<.001) and a greater engagement in online mental health communication (*β* range=.31-.36; *P*<.001). Those with depression and anxiety also had a greater likelihood of using digital mental health tools (depression OR 2.02; *P*=.001; and anxiety OR 1.71, *P*=.005) and a greater engagement in online mental health communication (depression *β*=.21; *P*<.001; and anxiety *β*=.16; *P*<.001). Neither anxiety nor depression was a significant moderator.

**Conclusions:**

Exposure to online racism, digital mental health tools use, and online mental health communication are linked for Black young adults. Black young adults may use digital mental health tools and engage in mental health communication online when they experience online racism or may experience online racism when they use these tools and engage online, necessitating further longitudinal analyses of these relationships. Complementary digital intervention implementation strategies that support mental health while mitigating online racism are needed. Ensuring that digital tools and mental health communications opportunities are safe, culturally relevant, and free from online racism is a set of mutually reinforcing priorities for digital interventions.

## Introduction

Given their convenience and accessibility, digital mental health tools and communication about mental health online are popular among young adults (aged 18‐29 y) [1], who are *almost constantly* online [2] to connect with others and seek advice [3]. Broadly, digital mental health tools are technologies (eg, apps, online services, and teletherapy) that provide support for mental health symptoms [4-6] and are used by those with [7] and without [8] formal mental health diagnoses. Young adults may also engage in online mental health communication (online engagement with mental health–related content and conversations), such as joining a mental health–related online group and sharing personal mental health experiences [8,9]. Given the early onset and prevalence of anxiety and depression in young adulthood [10], understanding how young adults use digital mental health tools and engage in online mental health communication can inform digital mental health interventions for this population.

Black young adults who experience mental health challenges may particularly benefit from the convenient and accessible use of digital mental health tools and online mental health communication due to the barriers they face to receiving traditional mental health care (eg, financial factors, stigma, and discrimination) [11]. At the same time, Black young adults may face negative race-related experiences while they use digital mental health tools and engage in online mental health communication. One such experience is exposure to anti-Black racism (hereafter racism), defined as a sociopolitical system that manifests in differential and negative treatment of and cultural messages about Black individuals [12]. Racism is easily disseminated in digital and online spaces due to a *digital freedom of speech* afforded to users, where engagement in racist acts may be more overt than in nonanonymous, offline settings [13]. For Black young adults, exposure to online racism can entail not only being the direct target of interpersonal racist attacks (eg, receiving negative comments or threats online because one is Black) but also vicariously witnessing racist interactions online or being exposed to derogatory content about Black individuals (eg, user comments calling a dark-skinned Black woman in a video ugly or hypersexual) [13-15]. Some of these vicarious and second-hand exposures involve traumatic content, such as images and videos of people being assaulted or even killed [16,17]. Aligned with a racial trauma framework [18], exposure to online racism is stressful, threatening one’s sense of self and emotional and social well-being [19-21]. Therefore, such exposure may be associated with the degree to which Black young adults use digital mental health tools and engage in online mental health communication.

Although much of the research has focused on social media specifically, the theory of online and digital spaces as both a stressor and a coping tool [22] suggests that digital mental health tools and online mental health communication could be engaged as ways of coping with the stressor of online racism. Many Black young adults go online to seek support [23] and find racially affirming social connections [24,25]. Therefore, the use of digital mental health tools and online mental health communication may help Black young adults proactively create and sustain networks of social support—emotional, instrumental, and informational—that can be engaged in reaction to race-related challenges they may face [21,26,27]. For instance, exposure to online racism for Black young adults may promote increased engagement in antiracist online spaces [28,29], such as online support groups, thereby potentially increasing communication with others about emotionally impactful and stressful racist content and interactions. To the extent that digital mental health tools and engagement in mental health communication online are believed to be effective mental health supports during times of stress, more frequent exposure to online racism may be associated with greater use.

Conversely, perhaps using some digital mental health tools or engaging in online mental health communication is itself associated with more exposure to online racism. In this case, Black young adults may not be using these digital resources to cope, but rather, more frequent exposure to online racism may be an unfortunate but unavoidable part of their digital experience. Research and theory on the health impacts of digital platforms and online contexts suggest that online racism is a structural part of many technologies, including educational tools and resources, which may perpetuate denigrating or stereotypical ideas about Black people in their design choices and content [15]. In this way, Black young adults who use digital mental health tools and engage in more frequent online mental health communication may face greater exposure to online racism. The theory of online and digital spaces as both a stressor and a coping tool [22] also suggests that online racism could be an additional potential source of stress in an already stressful, racist digital environment. Negative experiences in digital spaces may reduce digital engagement more broadly due to concerns about increased exposure to negative content or social interactions. Exposure to online racism is one type of negative interaction online that can cause stress [22]. Such stress may lead some Black young adults to disengage as a means of coping to preserve mental health [30]. To the extent that digital mental health tools and online mental health communication are seen as a part of a unified digital landscape, Black young adults’ more frequent exposure to online racism could be associated with less use of these tools or engagement.

Associations between exposure to online racism, digital mental health tool use, and online mental health communication engagement may also differ for those with versus without depression or anxiety. In general, those with depression or anxiety use digital mental health tools, including mental health forums, websites, apps, and crisis lines, at higher rates than those without [31]. However, these tools and general engagement in communication about mental health online are also used by the general public to support their mental health and manage stress and negative emotions as well [32]. Black young adults with depression or anxiety may show higher use or engagement compared to Black young adults without these symptoms due to having more frequent or severe symptoms of depression or anxiety than Black young adults without these conditions. The severity or frequency of symptoms may mean that they experience relatively more psychological distress from exposure to racism [11]. Whether exposure to online racism is associated with use of digital mental health tools and engagement in online mental health communication differently for Black young adults with versus without depression or anxiety remains an empirical question. In this study, we examine moderation by depression or anxiety to inform the degree to which implementation and intervention efforts focused on digital mental health tools and resources should be tailored by mental health status.

This study aimed to describe whether Black young adults’ use of digital mental health tools and frequency of engagement in online mental health communication are associated with exposure to online racism. We also examined whether associations between online racism and digital mental health tool use and engagement in online mental health communication differed for those with depression or anxiety. To investigate these aims, we conducted an initial exploratory investigation, as several distinct hypotheses could be drawn from the extant literature, and we acknowledge the potential bidirectional nature of the findings. As digital mental health resources are growing in popularity, such initial descriptive research can elucidate considerations for seeking digital mental health tools among Black young adults using digital and online technologies.

## Methods

### Procedures

Data came from a larger investigation of mental health and help-seeking conducted via the completion of an online cross-sectional survey from July to September 2024. As the purpose of the larger study was to understand the mental health of Black and Asian American, Native Hawaiian, or Pacific Islander young adults, we included anyone aged 18 to 29 years who self-identified as Black American or Asian American and resided in the United States. Potential participants were asked, “Please describe your race or ethnicity (select all that apply): American Indian or Alaska Native; Asian or Native Hawaiian/Pacific Islander; Black or African American; Hispanic or Latino; Middle Eastern or North African; White.” Individuals who selected Black or African American or Asian or Native Hawaiian or Pacific Islander were included in the study.

Participants were recruited online through Qualtrics Panels, a service through which prequalified individuals complete surveys for research purposes, generating a nonprobability online convenience sample. Quotas were implemented for gender, education, and geographic region to ensure sufficient representation of these factors among survey participants. The validity of Qualtrics panel data is comparable to a traditionally recruited community sample [33]. Each participant panel has its own confirmation procedures to ensure participant verification and data quality. All panel partners verify respondent address, demographic information, and email address using third-party validation platforms (eg, TrueSample, RelevantID, and USPS). Qualtrics Panels removed those who completed the survey too fast, straight-line responders, and multiple responders from the same IP addresses. The online survey also included a reCAPTCHA to detect potential nonhuman responses and 2 attention check questions. Participants who failed these were excluded. With these rigorous data quality standards, only 40% of initiated responses were able to qualify.

### Measures

#### Use of Digital Mental Health Tools

Participants indicated whether they had tried to get help from digital tools for problems with their mental health or emotions in the past year, using items developed for the survey. They were provided with a list of digital tools and asked to select all that they used, including (1) wellness apps or websites; (2) apps or websites for treating depression, anxiety, or other mental health conditions; (3) online or telephone-based therapy services; and (4) online support groups. They were also given additional space to write a description of other tools (eg, watching YouTube videos for mental health information). Participants’ selected responses were coded into a dichotomous variable, with 0 representing no reported use of any tools and 1 representing reported use of one or more tools, including those that participants wrote into the textbox.

#### Online Mental Health Communication

Participants indicated how often they had communicated about mental health online in the past year using 4 items developed for the survey. The items included (1) joining forums or closed social media groups on specific mental health topics, (2) doing hashtag searches for mental health topics on social media, (3) following people online who share about mental health conditions, and (4) sharing about their own poor mental well-being online. Participants indicated the frequency of each item using a Likert-type response scale from 0 (*never*) to 4 (*once a week or multiple times a week*). The mean score was computed from the items to represent the overall frequency of online mental health communication, such that a higher score indicates more frequent online mental health communication. The internal consistency of the scale was acceptable (sample *α*=.87).

#### Exposure to Online Racism

The Perceived Online Racism Scale-Very Brief [34] was used to measure exposure to online racism. Participants indicated how often they had experienced 6 items online in the past 6 months, using a Likert-type scale, ranging from 1 (*never*) to 5 (*always*). Example items include “seen online videos that portray my racial/ethnic group negatively” and “received posts with racist comments.” A mean score was calculated such that higher scores indicated more frequent exposure to online racism. The internal consistency of the scale was acceptable (sample *α*=.90).

#### Depression or Anxiety

Depression was measured using the Patient Health Questionnaire-9 (PHQ-9) [35]. Participants indicated how often they had been bothered by 9 common depressive symptoms over the past 2 weeks on a Likert-type scale from 1 (*not at all*) to 4 (*nearly every day*). Example items include “little interest or pleasure in doing things” and “feeling down, depressed, or hopeless.” Participants’ responses were scored by computing the sum of items. The internal consistency of the scale was acceptable (sample *α*=.90). Continuous scores were dichotomized to correspond to clinical cutoffs for major depressive disorder. Depression not indicated (PHQ-9 score <10) was coded as 0. Depression indicated (PHQ-9 score ≥10) was coded as 1. Anxiety was measured using the General Anxiety Disorder 7-item scale (GAD-7) [36]. Participants indicated how often they had been bothered by 7 common anxiety symptoms over the past 2 weeks on a Likert-type scale from 1 (*not at all*) to 4 (*nearly every day*). Example items included “feeling nervous, anxious, or on edge” and “not being able to stop or control worrying.” Participants’ responses were scored by computing the sum of items. The internal consistency of the scale was acceptable (sample *α*=.92). Continuous scores were dichotomized to correspond to clinical cutoffs for generalized anxiety disorder. Anxiety not indicated (GAD-7 score <10) was coded as 0. Anxiety indicated (GAD-7 score ≥10) was coded as 1. Having depression (yes or no) and having anxiety (yes or no) were 2 separate variables in our analyses.

#### Analytic Covariates

Age, gender, and internet use were included as covariates. Age was reported in the number of years. On the basis of participant responses, there were three categories of gender identity present in the sample: woman; man; and transgender, fluid, or nonbinary. Participants were asked to report their internet use by indicating how often they used the internet on a typical day, with response options from 0 (*not at all*) to 4 (*almost constantly*).

### Analysis Plan

We first examined the presence of missing data. Participants in the analytic sample had missing data on the following variables: use of digital mental health tools (n=50, 4.98%) and gender (n=8, 0.80%). Taken together, 57 (5.67%) participants had any missing data. Those with missing data had lower daily internet use (*t*_59.19_=2.34; *P*=.02), did not have consistent internet access (*χ*^*2*^_1_=10.3; *P*=.001), and reported less exposure to online racism (*t*_1003_=2.68; *P*=.007). There were no differences between those with or without missing data on outcome variables by geographic region (*χ*^*2*^_3_=0.5), age (*t*_1003=_1.58; *P*=.12), frequency of engagement in online mental health communication (*t*_1003_=1.15; *P*=.25), depression (*χ*^*2*^_1_=0.3), or anxiety (*χ*^*2*^_1_=0.3). In sensitivity analyses, we also ran our analytic models excluding those with missing data. Results did not differ.

We first conducted descriptive statistics to describe exposure to online racism, the use of digital mental health tools, engagement in online mental health communication, and rates of depression or anxiety in our sample. Next, we conducted 2 separate path analysis regression models using maximum likelihood estimation, which were each run in Mplus. Path analysis models can handle multiple outcome variables in each model, including outcome variables of different types (eg, binary, continuous). Our model 1 included predictors of online racism and *depression,* and the interaction between the two, on outcomes of use of digital mental health tools (modeled with logistic regression) and engagement in online racism (modeled with linear regression). Our model 2 included predictors of online racism and *anxiety,* and the interaction between the two, on outcomes of use of digital mental health tools (modeled with logistic regression) and engagement in online racism (modeled with linear regression). As depression and anxiety were relatively strongly correlated (*r*_1003_=0.61; *P*<.001), we included these variables in separate models and used a Bonferroni α adjustment (*α*=.05 divided by 2 models for a corrected *α*=.025) to account for artificial type 1 error inflation due to multiple comparisons [37]. In this way, a *P* value less than .03 for any given model parameter was considered significant. If the interaction term (ie, multiplication of online racism and depression or anxiety) was significant, this was evidence of moderation. Exposure to online racism was grand mean centered, and the interaction term was computed using this mean-centered variable. These path analysis models were fully saturated (ie, all possible parameters were estimated), such that model fit indices were not a consideration.

A conservative post hoc power analysis [38] for the most complex model effect (ie, interaction term) using the smallest observed correlations between model variables, observed variable alpha reliabilities, and a Bonferroni-adjusted critical α level of .025 indicated that our power was .99 with a sample size of 1005.

### Ethical Considerations

The study was approved by the University of Southern California Institutional Review Board (APP-24‐05114; principal investigator: HO). All participants provided informed consent before completing the study, and all data were deidentified before data analysis. Participants who completed the survey received compensation based on their preference, such as cash, airline miles, and vouchers.

## Results

### Participant Demographics

Our research questions are focused on Black young adults. Therefore, the analytic sample for the current investigation included 1005 monoracial Black young adults aged 18 to 29 (mean age 24.07, SD 3.04) years from the larger study (Table 1). Although multiracial Black young adults were included in the larger study from which the data are drawn, we only included monoracial Black young adults in the current investigation because their experiences of online racism are likely to differ compared to those of their multiracial Black peers. There were approximately equal numbers of women (504/1005, 50.6%) and men (490/1005, 49.1%) in the sample. Most participants resided in the southern United States (481/1005, 47.9%) and had health insurance (89%). A majority indicated that they were almost constantly on the internet (610/1005, 60.7%) and had internet access at home or work (988/1005, 98.3%).

**Table 1.** Characteristics of the analytic sample of Black monoracial young adults (N=1005).

Characteristic	Values
Age, mean (SD), years	24.07 (3.04)
Gender, n (%)	
Woman	504 (50.6)
Man	490 (49.1)
Transgender, nonbinary, or fluid	3 (0.3)
Internet use, n (%)	
Not at all	9 (0.9)
Less than a few times a day	25 (2.5)
A few times a day	107 (10.6)
Many times a day	254 (25.3)
Almost constantly	610 (60.7)
US region, n (%)	
Midwest	203 (20.2)
Northeast	235 (23.4)
South	481 (47.9)
West	86 (8.6)
Health insurance, n (%)	
No	111 (11)
Yes	894 (89)
Internet access at home or work, n (%)	
No	17 (1.7)
Yes	988 (98.3)
Exposure to online racism, mean (SD)	2.58 (1.10)
Use of digital mental health tools, n (%)	
None	437 (45.8)
One or more	518 (51.5)
Online mental health communication, mean (SD)	1.21 (1.15)
Depression, n (%)	
No	508 (50.5)
Yes	497 (49.5)
Anxiety, n (%)	
No	579 (57.6)
Yes	426 (42.4)

### Descriptive Statistics

Most of the sample (891/1005, 88.66%) reported any exposure to online racism in the past 6 months. On average, exposure occurred between ‘sometimes’ and ‘half the time’ in the past 6 months (mean 2.58, SD 1.10). This mean frequency was comparable to that in other studies using the same measure [39]. Approximately half (518/1005, 51.5%) of the sample reported using 1 or more digital mental health tools in the past year, while the remainder reported no use of digital mental health tools. On average, participants engaged in online mental health communication less than once a month (mean 1.21, SD 1.15). On the basis of their PHQ-9 and GAD-7 scores, approximately half (497/1005, 49.5%) of the participants in the sample were classified as having depression, and approximately 40% (426/1005) were classified as having anxiety. Thirty-six percent (362/1005) of participants had both depression and anxiety. Bivariate correlations among study variables are presented in Table 2.

**Table 2.** Bivariate correlations among study variables.

Variable	Internet use	Age	Gender	Online racism	Depression	Anxiety	DMHT^a^	OMHC^b^
Internet use	—^c^	—	—	—	—	—	—	—
Age	.04	—	—	—	—	—	—	—
Gender	.05	−.08^d^	—	—	—	—	—	—
Online racism	.01	−.03	.03	—	—	—	—	—
Depression	−.10^e^	−.04	.04	.35^f^	—	—	—	—
Anxiety	−.04	−.04	.05	.33^f^	.61^f^	—	—	—
DMHT^a^	.07*	−.02	−.09^e^	.29^f^	.25^f^	.20^f^	—	—
OMHC^b^	−.08^e^	.02	−.03	.42^f^	.32^f^	.28^f^	.57^f^	—

a DMHT is the use of digital mental health tools.

b OMHC is engagement in online mental health communication.

c Not applicable.

d *P*<.05.

e *P*<.01.

f *P*<.001.

### Path Analysis

Exposure to online racism was associated with the greater use of digital mental health tools and more frequent online mental health communication, controlling for depression and anxiety (Multimedia Appendix 1). In both models 1 and 2, more frequent exposure to online racism was associated with a significantly increased likelihood of using digital mental health tools (model 1, odds ratio [OR] 1.72, 95% CI 1.42-2.08; *P*<.001; and model 2, OR 1.84, 95% CI 1.53-2.21; *P*<.001). More frequent exposure to online racism was also associated with more frequent online mental health communication (model 1, β=.31, 95% CI 0.23-0.39; *P*<.001; model 2, *β*=.36, 95% CI 0.29-0.44; *P*<.001).

Controlling for online racism, having depression or anxiety was also positively associated with the use of digital mental health tools and online mental health communication. Black young adults who exhibited clinically significant depressive symptoms were more likely to report using digital mental health tools (OR 2.02, 95% CI 1.52-2.69; *P*=.001) and reported more frequent engagement in online mental health communication (*β*=.21, 95% CI 0.15-0.27; *P*<.001). Similarly, Black young adults who exhibited clinically significant symptoms of anxiety were 71% more likely to use digital mental health tools (OR 1.71, 95% CI 1.28-2.28; *P*=.005) and were more frequently engaged in online mental health communication (*β*=.16, 95% CI 0.10-0.22; *P*<.001). The association between exposure to online racism and use of digital mental health tools and frequency of online mental health communication did not significantly differ for those who had depression or anxiety compared to those who did not (see for nonsignificant interaction statistics).

## Discussion

### Principal Findings

As members of a generation that grew up with digital technologies, Black young adults are especially likely to use digital mental health tools and engage in online mental health communication. Despite this, a majority of the existing literature on digital mental health tools and online mental health communication among young adults has tested associations in predominantly White samples [6,8,9]. This body of literature has also neglected the role of negative race-related online experiences in shaping the use of digital mental health resources, leaving gaps in our understanding of the unique digital mental health–related experiences of Black young adults. As digital and online mental health solutions continue to be developed as promising and accessible new treatments for youth [6], this study directly informs the yet unexplored role of online racism in how Black young adult populations seek digital mental health resources and provides design and research implications for such solutions for this population.

More frequent exposure to online racism was associated with greater digital mental health tool use and more frequent online mental health communication. Although we cannot know the degree to which Black young adults used tools or engaged online in response to online racism specifically, our findings align with research suggesting that Black young adults may engage with digital tools in an attempt to cope with racism-related stress or protect their well-being. For instance, when engaging on social media apps, Black young adults curate their online environment by filtering content, participate selectively in culturally affirming communities, and use engagement tools such as hashtags to foster safety and connection [40-42]. Black young adults may use digital mental health tools when experiencing more frequent online racism because they perceive these tools to be helpful. This assertion is aligned with evidence that attitudes toward digital mental health solutions are positive, that behavioral control is high, and that there is already a subjective norm of using these tools among young adults [32,43]. Black young adults are also likely to share personal experiences about their mental health from stressful, negative experiences in online spaces such as support groups [29]. Although engagement in online mental health communication was relatively low in the sample, it may be that more frequent exposure to online racism is a stressor that catalyzes this engagement as a coping response [28,30]. Conversely, it could be that those who use digital mental health tools or engage more frequently in online mental health communication are exposed to more online racism in these digital contexts. Although it has been theorized that exposure to online racism can be embedded in any technological creation [15], most research examines social media rather than specific mental health tools and resources. The degree to which exposure to online racism in digital mental health content and resources particularly impacts their use remains an important future direction for research. Another related potential explanation for our findings is that Black young adults may perceive a distinction in the contexts in which online racism occurs and the contexts in which they use mental health resources and engage in online mental health communication. If their online racism exposure is occurring primarily on social media, using a wellness app (eg, Headspace) would not necessarily increase their exposure to online racism. In this way, Black young adults may flexibly draw on digital mental health tools and resources that they feel are safer during times of stress.

Exposure to online racism was associated with more digital mental health engagement regardless of clinical levels of anxiety or depression. Higher psychological distress has been associated with greater use of online mental health programs among young adults [44]. Yet some studies find no evidence that baseline symptom severity affects digital mental health app use [45]. In this way, perhaps Black young adults turn to digital mental health tools and engagement in online mental health communication when they feel stress and sadness, including from online racism [16], intending to remedy the negative emotions from these experiences. This finding suggests that online racism may be associated with the use of digital mental health tools and engagement in online mental health communication for a wide swath of Black young adults, even if they do not meet the diagnostic criteria for depression or anxiety, signaling the potential reach and utility of these digital mental health resources for this population. Although the association between exposure to online racism and digital mental health resources did not vary by depression or anxiety, Black young adults with these conditions used digital mental health tools at higher rates and engaged in more online mental health communication compared to those without depression or anxiety. This finding is supported by literature that suggests young adults experiencing mental health challenges are more likely to use online mental health–related resources [8]. This study extends this body of research by examining digital mental health tools and online mental health communication among Black young adults with anxiety or depression.

### Limitations and Future Directions

Our study is not without limitations. Although the role of online racism as a stressor impacting behavioral outcomes, rather than the reverse, has been theorized [46] and documented [47], we are unable to infer causality or test the directionality of variables in this cross-sectional correlational study. It may be that those more likely to use digital mental health tools and engage in online mental health communication may experience online racism, or vice versa. Future research should examine these variables using longitudinal designs to measure use and engagement specifically in response to both general stress and specific stressors such as online racism. For instance, a daily diary study assessing the frequency of online racism experienced and the use of digital mental health tools and engagement in online mental health communication in response to such experiences on a given day could provide promising insights regarding Black young adults’ reliance on online support resources when experiencing online racism. Such findings could further establish a temporal relationship showing that when experiencing online racism, Black young adults may turn to online social support resources (ie, digital mental health tools and online mental health communication) as coping strategies. This could have implications for the expansion and development of culturally tailored digital mental health tools and online mental health communication mechanisms (eg, a private online support group for experiencing online racism) specific to the needs of Black young adults. Second, although the time frame of measures of online racism (ie, past 6 mo) and outcomes (ie, past year) is appropriate for these constructs, they are assessed via self-report measures that may be affected by recall bias. Additionally, our measure of digital mental health tools was broad, with usage measured as a binary indicator. Although this approach allowed us to generate initial evidence for associations as a starting point for future research, it did not capture nuanced patterns of usage, preferences, or satisfaction. Previous literature has suggested that young adults have preferences in features or types (ie, web-based or mobile self-help) of their digital mental health tools [8] that could be impacted by their level of care needs for mental health conditions, such as depression or anxiety. As Black young adults have tailored their online experiences, such as by using culturally affirming hashtags and engaging in online communities specific to members of their racial group [41,42], future research investigating distinct aspects of digital mental health resource use (eg, intensity of use, digital platform–specific effects, and types of digital mental health resources used) is integral and has implications for developing culturally relevant digital mental health interventions for Black young adults.

Another future direction is to investigate the types of online spaces (eg, social media platforms and online forums) where Black young adults use digital mental health tools and engage in online mental health communication. Research suggests that Black young adults may feel more comfortable discussing these experiences in anonymous communities than on social networked or public platforms such as Facebook feed [29]. Future work could explore how platform type, privacy, and identity shape help-seeking. Our sample was drawn using quota sampling; while this method enhances demographic balance, it remains a form of convenience sampling and is not probabilistic. Participants who enroll in online survey panels may differ systematically from those without the same internet access or who lack interest in online research participation, which limits generalizability. In this study, we focused on monoracial Black young adults, but the online racism experiences of multiracial Black young adults could differ, and we do not necessarily expect our results to generalize to this population. Future research that considers intersectionality and samples large enough numbers of Black multiracial populations to appropriately center on their unique experiences remains an important future direction. Finally, most participants were from the southern United States; however, as the availability of certain digital mental health tools varies by state [48], our results may not generalize to other regions. Our findings are focused on online racism and therefore do not capture intersectional experiences of discrimination (eg, gendered racism and racist ableism) that Black young adults may experience online. Further development or adaptation of measures is needed to contextualize such experiences and their impact on digital mental health resources and engagement.

### Conclusions

This study highlights the use of digital resources aimed at promoting mental health, such as digital mental health tools and online mental health communication, for Black young adults. Exposure to online racism was linked to greater use of digital mental health tools and more frequent online mental health communication. Mental health practitioners should be aware of the role that digital contexts play in shaping the experiences of Black young adults, both positive and negative, and consider asking about current use or suggesting tools and online mental health communication as resources for clients, when appropriate. At the same time, ensuring that these tools and opportunities for communication are safe, free of online racism, and culturally relevant remains an ongoing parallel priority for researchers, policymakers, and developers.

## Supplementary material

10.2196/80657 Multimedia Appendix 1 Results of regression analyses assessing associations with the use of digital mental health tools and online mental health communication (N=1005).

## Abbreviations

GAD-7: Generalized Anxiety Disorder-7
OR: odds ratio
PHQ-9: Patient Health Questionnaire-9
